# Analysis of Resonance Asymmetry Phenomenon in Resonant Fiber Optic Gyro

**DOI:** 10.3390/s18030696

**Published:** 2018-02-26

**Authors:** Zhuoyan Li, Nie He, Xuqiang Sun, Chao Jin, Chengxiang Liu, Xu Wu

**Affiliations:** 1 Sino-German College for Intelligent Manufacturing, Shenzhen Technology University, Shenzhen 518060, China; 2 Guangdong Provincial Key Laboratory of Micro/Nano Optomechatronics Engineering, College of Optoelectronic Engineering, Shenzhen University, Shenzhen 518060, China; 2160190403@email.szu.edu.cn (Z.L.); 2160190401@email.szu.edu.cn (N.H.); 2150190122@email.szu.edu.cn (X.S.); 2151190215@email.szu.edu.cn (C.J.); 3 Guangdong Provincial Key Laboratory of Micro/Nano Optomechatronics Engineering, College of Mechatronics and Control Engineering, Shenzhen University, Shenzhen 518060, China

**Keywords:** resonant fiber optic gyro, normal mode loss difference, resonance asymmetry

## Abstract

This experiment demonstrated the resonance asymmetry phenomenon in the resonant fiber optic gyro. The asymmetry of resonant curve affects the system open-loop bias and its stability, which directly affects the accuracy of angular velocity measurement. In this paper, a new mathematic model is established. The influence of the coupler normal mode loss difference (the phase difference between the coupler cross port output optical field and direct port is less than the ideal π/2) on the symmetry of resonant curve, the resonant signal modulated by the triangular wave, and the demodulation curve are analyzed. Moreover, the asymmetry of the resonant curve leads to the asymmetry of the resonant signal, as modulated by the triangular wave and the demodulation curve from the theoretical simulation and the experiment.

## 1. Introduction

Gyro is an inertial sensor for the rotation rate measurement [1]. At present, fiber optic gyro, micro-optic gyro, laser gyro has great development potential because of its own advantages [1,2,3,4,5,6,7,8]. Resonant fiber optic gyro (RFOG) based on the Sagnac effect has the advantages of high sensitivity and large dynamic range [2]. When compared with the traditional interferometric fiber optic gyro (IFOG), RFOG can achieve the same detection precision as IFOG with a much shorter fiber length, which gives evident superiority in the further integration, as well as improves its application value in the fields of navigation, aerospace, defense industries, and guidance systems [1,2,3,4,5,6,7,8,9].

RFOG is a kind of high-precision angular velocity sensor based on the optical Sagnac effect. It can detect the angular velocity information of the gyroscopic rotation by detecting the resonance frequency difference of the clockwise (CW) and counterclockwise (CCW) light waves that are caused by rotation. However, the optical Sagnac effect has an extremely weak effect in a RFOG system. The working frequency of the laser is as high as 193 THz. When the diameter of the optical fiber resonant ring of the RFOG system is 15 cm and the rotational angular velocity is 1 deg/s, the resonant frequency difference caused by rotation is only 116 Hz. Thus, detecting the signal of several hundred Hz at such a high working frequency is very difficult. Meanwhile, when the external environment, including temperature and stress changes, the laser’s center frequency and the resonant frequency of the fiber ring resonator (FRR) will also change. Therefore, directly measuring the resonance frequency difference of the CW and CCW light waves caused by the rotation using the RFOG system is extremely difficult; in addition, appropriate modulation and demodulation techniques are needed to improve the sensitivity of the signal detection system [9]. In a single closed-loop RFOG system, the triangular wave modulated signals with different frequencies are used to modulate the CW and CCW light waves. One of the light waves is processed by a field-programmable gate array (FPGA) based digital detection control circuit, whereas the FPGA output feedback signal controls the center frequency of the laser. Thus, the laser is locked into the resonant frequency of this light wave. Afterward, the demodulation curve of the output signal of the other light wave will reflect the resonant frequency difference of the two light waves to achieve the angular velocity measurement. If the resonant curve is asymmetric, the demodulation curve is asymmetric, which will result in the laser’s center frequency being unable to be locked to the resonance frequency of one of the light waves. Thus, this phenomenon affects the system open-loop bias and its stability, which directly affects the angular velocity measurement accuracy.

Stokes et al. reported a FRR composed of a single mode fiber for the first time in 1982 and found the resonance asymmetry in the resonant curve measurement [10]. They also explained and analyzed the resonant curve asymmetry in 1983 [2]. The normal mode loss difference of the coupler will result in the phase difference between the coupler cross port output optical field and the direct port is less than the ideal π/2. Thus, the resonant curve appears asymmetric phenomenon [10,11]. R. Carroll found that the phase difference between the cross port output optical field and the direct port in the coupler with low/high coupling coefficients is different from π/2. To form a high-resolution resonator, using coupler with low/high coupling coefficients is necessary [12]. A coupler with low/high coupling coefficients can also be used into a FRR of the resonant fiber optical gyro; thus, the phase difference between the coupler cross port output optical field and direct port is less than the ideal π/2, resulting in the asymmetry phenomenon of the resonant curve. Zhejiang University has also conducted an in-depth study on the asymmetry phenomenon of the resonant curve due to the normal mode loss difference of the coupler [13]. In a RFOG, the coupler factor is not the only factor that causes the asymmetry phenomenon of the resonant curve. Polarization fluctuations [14], optical Kerr effect [15], FRR ambient temperature fluctuations [16], backscattering [17], and other factors can cause the resonant curve to be asymmetric. However, the phase difference between the coupler cross port output optical field and direct port is less than the ideal π/2, which is the most important factor affecting the asymmetry of the resonant curve [13].

In this paper, a new mathematic model is established. The influence of the normal mode loss difference of the coupler (the phase difference between the coupler cross port output optical field and direct port is less than the ideal π/2) on the symmetry of the resonant curve, the resonant signal modulated by the triangular wave, and the demodulation curve are all analyzed in detail. At the same time, the asymmetry of the resonant curve will lead to the asymmetry of the resonant signal modulated by the triangular wave and the demodulation curve from the theoretical simulation and the experiment.

## 2. System Structure of Resonant Fiber Optic Gyro 

**Figure 1:**
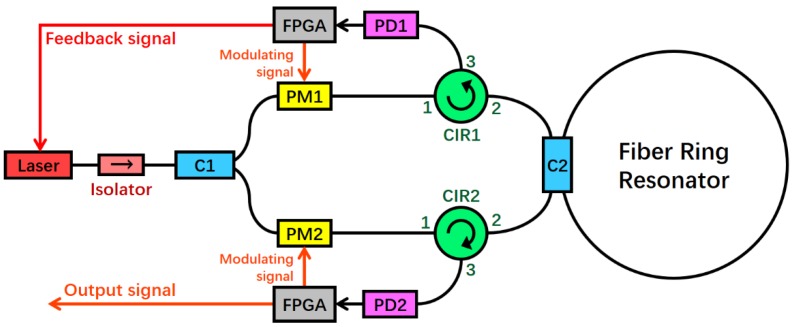
System structure of RFOG. (FPGA: field-programmable gate array, PD: photodiode, PM: phase modulator, C: coupler, and CIR: circulator).

RFOG system structure is depicted in Figure 1. The laser light emitted by a narrow-line-width semiconductor laser is divided into two equal power beams by a 50:50 coupler C1 after passing through an isolator. The two light beams passed through the LiNbO_3_ phase modulator (PM)1 and PM2, respectively, which are modulated by the modulated signals of different frequencies. Afterwards, the two beams are incident to the FRR through the circulators (CIR), namely, CIR1 and CIR2, in the CCW and CW directions, respectively. C2 is the coupler connected to the FRR. Finally, the two light beams are output to photodiode (PD) 2 and PD1 via CIR2 and CIR1, respectively. Among the light waves, the CW light wave is processed by the FPGA-based digital detection control circuit. Afterward, the FPGA output feedback signals to control the center frequency of laser; thus, the laser is locked into the resonant frequency of the CW light wave. At this moment, the output optical signal of the CCW light wave can reflect the resonance frequency difference of the CW and CCW light waves. Moreover, the rotational angular speed can be obtained after being processed by FPGA.

## 3. Model Establishment and Simulation Results

### 3.1. Resonant Curve

**Figure 2:**
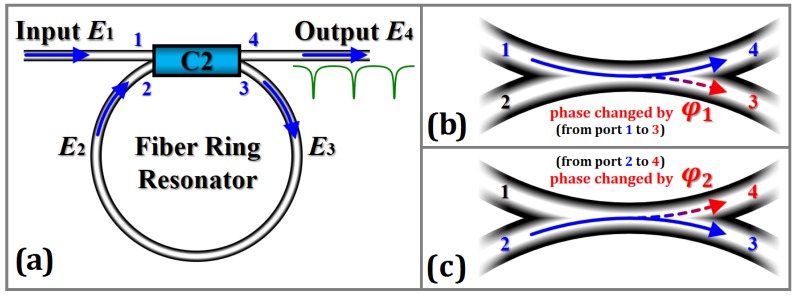
(**a**) Transmission process of one of the light waves in FRR. (**b**) The phase is changed by *φ*_1_ from port 1 to 3. (**c**) The phase is changed by *φ*_2_ from port 2 to 4. (*E_i_*: the optical field complex amplitude of port *i* is denoted).

The transmission process of one of the light waves in the FRR is depicted in Figure 2a. The incident light wave is divided into two beams from the Port 1 through the coupler. A light wave is directly output from Port 4, whereas the other light wave enters the FRR from Port 3. After propagating a circle, the light wave enters the coupler again through Port 2. A part of the light wave is output through Port 4, while the remaining part of the light wave enters the FRR again from Port 3 and continues to propagate in the cavity. Using analogy, when the light wave propagates one circle into the resonator, a part of the light wave is output from Port 4 of the coupler C2, and the light waves output from Port 4 are superposed on each other. Multiple beam interference occurs. Moreover, the optical field complex amplitude and the light intensity of Port 4 are the results of multiple beam interference. If the optical field complex amplitude of port *i* is denoted by *E_i_* (where *i* = 1, 2, 3, 4), then the ratio of the optical field complex amplitude of Port 4 to the incident light is *E*_4_/*E*_1_ , while the ratio of the light intensity of Port 4 to the incident light is |*E*_4_/*E*_1_|^2^.

If the light passes through the cross port of the coupler C2, the phase changes by π/2. When the light wave passes through the cross port to reach the inside of FRR or passes through the cross port to reach the outside of FRR, the phases both change by π/2. Multiple beam interference occurs among the light waves arriving at Port 4 through the direct port and the light in the FRR that is constantly coupled to Port 4. Only when the light satisfies a certain optical frequency, then it can result to the resonance phenomenon. Furthermore, the output curve of Port 4 presents a downward depression (power is 0 or small). Thus, if |*E*_4_/*E*_1_|^2^ takes a minimum value, then it meets the FRR resonance conditions.

However, if the light wave passes through the cross port of coupler C2, the phase change is less than the ideal π/2. To further discuss the effect of the phase difference between the output optical field of the coupler cross port and the direct port being less than the ideal π/2 on the output optical power, we need to analyze the simultaneous equations satisfied by the optical field of each port and solve the ratio of the optical field complex amplitude of Port 4 to the incident light *E*_4_/*E*_1_ and the ratio of the light intensity of Port 4 to the incident light |*E*_4_/*E*_1_|^2^. As shown in Figure 2b, c, the light phase from Port 1 to Port 3 is changed by *φ*_1_, and the light phase from Port 2 to Port 4 is changed by *φ*_2_. The optical power coupling ratio between the coupler direct port and cross port is *k*:(1 − *k*). The remaining percentages of the optical power that is caused by insertion loss of the coupler and transmission loss of the fiber ring are *ρ*_c_ and *ρ*_L_, respectively. The light wave frequency entering the FRR is *ν*. The time required to enter the ring fiber resonator from Port 3 to propagate 1 circle to reach Port 2 is *τ*. The *q*-th resonant frequency is *ν_q_*, that is, *ν_q_* = *q*/*τ*. When the light wave frequency entering the FRR satisfies *ν* = *ν_q_*, the light wave resonates in the FRR. Then, the output optical field complex amplitude of each port can be expressed as

(1) $$E_{2}=\sqrt{\rho_{L}}e^{-j2\pi \nu \tau }E_{3},$$

(2) $$E_{3}=e^{j\varphi_{1}}\sqrt{\left(1-k\right)\rho_{c}}E_{1}+\sqrt{k\rho_{c}}E_{2},$$

(3) $$E_{4}=\sqrt{k\rho_{c}}E_{1}+e^{j\varphi_{2}}\sqrt{\left(1-k\right)\rho_{c}}E_{2}.$$

Combining Equation (1) to Equation (3), the ratio of the optical field complex amplitude from Port 4 to Port 1 can be expressed as

(4) $$\frac{E_{4}}{E_{1}}=\sqrt{k\rho_{c}}+\frac{e^{j\varphi_{0}}\left(1-k\right)\rho_{c}}{\sqrt{\rho_{L}^{-1}}e^{j2\pi \nu \tau }-\sqrt{k\rho_{c}}},$$

where *φ*_0_ = *φ*_1_ + *φ*_2_. The left and right sides of Equation (4) are modeled and then squared. In addition, the optical power ratio from Port 4 to Port 1 can be expressed as

(5) $$I={\left|\frac{E_{4}}{E_{1}}\right|}^{2}=\rho_{c}\left[k+\left(1-k\right)\sqrt{\rho_{c}\rho_{L}}\cdot \frac{2\sqrt{k}\cos\left(2\pi \nu \tau -\varphi_{0}\right)+\sqrt{\rho_{c}\rho_{L}}\left(1-k-2k\cos\varphi_{0}\right)}{1+k\rho_{c}\rho_{L}-2\sqrt{k\rho_{c}\rho_{L}}\cos2\pi \nu \tau }\right],$$

where *I* is the normalized intensity. If the light passes through the cross port of coupler C2, the phase changes by π/2, that is, *φ*_1_ = *φ*_2_ = π/2. Then, the ratio of the optical field complex amplitude from Port 4 to Port 1 can be expressed as

(6) $$\frac{E_{4}}{E_{1}}=\sqrt{k\rho_{c}}-\frac{\left(1-k\right)\rho_{c}}{\sqrt{\rho_{L}^{-1}}e^{j2\pi \nu \tau }-\sqrt{k\rho_{c}}}.$$

Similarly, the left and right sides of Equation (6) are modeled and then squared. The optical power ratio from Port 4 to Port 1 can be expressed as

(7) $$I={\left|\frac{E_{4}}{E_{1}}\right|}^{2}=\rho_{c}\left[1-\frac{\left(1-k\right)\left(1-\rho_{c}\rho_{L}\right)}{1+k\rho_{c}\rho_{L}-2\sqrt{k\rho_{c}\rho_{L}}\cos2\pi \nu \tau }\right].$$

**Figure 3:**
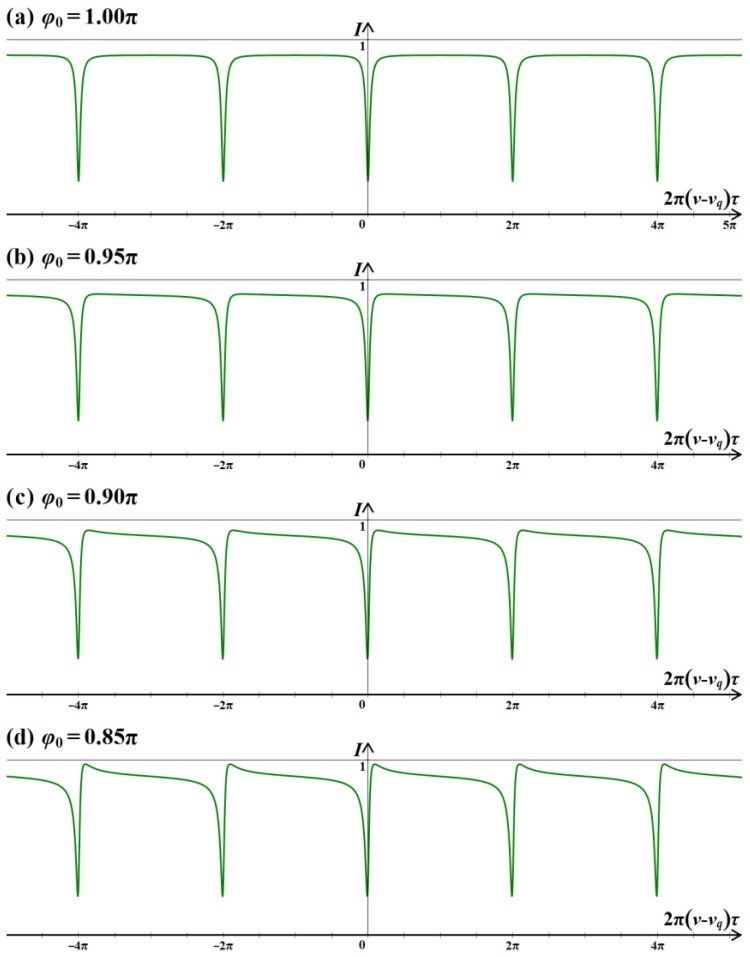
Simulated resonant curve of *φ*_0_ = (**a**) 1.00 π, (**b**) 0.95π, (**c**) 0.90π, and (**d**) 0.85π.

On the basis of the analysis above on the transmission process of light waves in FRR, the resonant curve can be simulated. The main simulation parameters are as follows: the splitting ratio and insertion loss of coupler C2 are 95:5 and −0.4 dB, respectively; the FRR transmission loss is −0.2 dB. If the light passes through the cross port of coupler C2, then the phase is changed by π/2, that is, *φ*_0_ = π. Thus, the resonant curve is a signal with symmetry, as shown in Figure 3a. If the light wave passes through the cross port of coupler C2, then the phase change is less than the ideal π/2, that is, *φ*_0_ ≠ π. Thus, the resonant curve is a signal with asymmetry. Figure 3b–d lists the cases of *φ*_0_ = 0.95π, 0.90π, and 0.85π, respectively. The results showed that the resonant curve is not symmetrical. The reason is that the light passing through the coupler C2 is not orthogonal and the phase change that is caused by non-orthogonal coupling mode is less than the ideal π/2, which will directly cause the resonant curve to be asymmetric. Moreover, the degree of asymmetry of the resonant curve is related to the degree of *φ*_0_ that deviates from π. The greater the degree of *φ*_0_ that deviates from π, the greater the degree of asymmetry of the resonant curve.

### 3.2. Modulation and Demodulation

Suppose the center frequency of the laser output light wave is *ν*_L_, that is, the center frequency of the light wave that is not modulated is *ν*_L_, and the initial phase is *ψ*_1_. Then, its complex amplitude can be expressed as

(8) $$E\left(t\right)=E_{0}e^{j\left(2\pi \nu_{L}t+\psi_{1}\right)}.$$

The frequency of the light wave incident to the FRR at this time is *ν* = *ν*_L_. Suppose the presence of a modulation signal, whose amplitude, frequency, and initial phase are *V*_p_, *f* and *ψ*_0_, respectively. This modulation signal is loaded into the phase modulator. Afterward, the light wave is modulated by the phase modulator, *ν* ≠ *ν*_L_. The complex amplitude of the modulated light wave can be expressed as

(9) $$E\left(t\right)=E_{0}e^{j\left[2\pi \nu_{L}t+\psi_{1}+\frac{\pi }{V_{\pi }}V_{p}P\left(2\pi ft+\psi_{0}\right)\right]},$$

where *V*_π_ is the half-wave voltage of the phase modulator, and P(∙) is a phase modulation function. When the resonant signal is modulated by the triangular wave,

(10) $$P\left(x\right)=\frac{2}{\pi }\arcsin\left(\sin x\right).$$

The differential of P is

(11) $$\frac{dP\left(x\right)}{dx}=P^{\prime }\left(x\right)=\frac{2}{\pi }\mathrm{sgn}\left(\cos x\right),$$

where sgn(∙) is a sign function. The laser’s frequency is changed slowly to let *ν*_L_ to vary slowly and linearly over time *t*. The complex amplitude of the light wave that has been modulated can be expressed as

(12) $$E\left(t\right)=E_{0}e^{j\left[2\pi \int \nu_{L}\left(t\right)dt+\frac{\pi }{V_{\pi }}V_{p}P\left(2\pi ft+\psi_{0}\right)\right]}.$$

The frequency *ν* of the light wave incident to the FRR at this time can be expressed as

(13) $$\nu =\frac{1}{2\pi }\cdot \frac{d}{dt}\left[2\pi \int \nu_{L}\left(t\right)dt+\frac{\pi }{V_{\pi }}V_{p}P\left(2\pi ft+\psi_{0}\right)\right]=\nu_{L}\left(t\right)+\frac{\pi }{V_{\pi }}V_{p}fP^{\prime }\left(2\pi ft+\psi_{0}\right).$$

Combining Equations (13) and (5), the optical power ratio of Port 4 to Port 1 can be expressed as

(14) $$I={\left|\frac{E_{4}}{E_{1}}\right|}^{2}=\rho_{c}\left\{k+\left(1-k\right)\sqrt{\rho_{c}\rho_{L}}\cdot \frac{2\sqrt{k}\cos\left\{2\pi \left[\nu_{L}\left(t\right)+\frac{\pi }{V_{\pi }}V_{p}fP^{\prime }\left(2\pi ft+\psi_{0}\right)\right]\tau -\varphi_{0}\right\}+\sqrt{\rho_{c}\rho_{L}}\left(1-k-2k\cos\varphi_{0}\right)}{1+k\rho_{c}\rho_{L}-2\sqrt{k\rho_{c}\rho_{L}}\cos\left\{2\pi \left[\nu_{L}\left(t\right)+\frac{\pi }{V_{\pi }}V_{p}fP^{\prime }\left(2\pi ft+\psi_{0}\right)\right]t\right\}}\right\}.$$

Equation (14) represents the expression that is satisfied by the phase-modulated resonant signal. By substituting Equation (11) into Equation (14), we can obtain the expression that is satisfied by the resonant signal modulated triangular wave, as follows:

(15) $$\begin{array}{c} I{|}_{\nu =\nu_{L}\left(t\right)+\frac{2}{V_{\pi }}V_{p}f\mathrm{sgn}\left[\cos\left(2\pi ft+\psi_{0}\right)\right]}\\ =\rho_{c}\left\{k+\left(1-k\right)\sqrt{\rho_{c}\rho_{L}}\cdot \frac{2\sqrt{k}\cos\left\{2\pi \left\{\nu_{L}\left(t\right)+\frac{2}{V_{\pi }}V_{p}f\mathrm{sgn}\left[\cos\left(2\pi ft+\psi_{0}\right)\right]\right\}\tau -\varphi_{0}\right\}+\sqrt{\rho_{c}\rho_{L}}\left(1-k-2k\cos\varphi_{0}\right)}{1+k\rho_{c}\rho_{L}-2\sqrt{k\rho_{c}\rho_{L}}\cos\left\{2\pi \left\{\nu_{L}\left(t\right)+\frac{2}{V_{\pi }}V_{p}f\mathrm{sgn}\left[\cos\left(2\pi ft+\psi_{0}\right)\right]\right\}t\right\}}\right\}. \end{array}$$

When the resonant signal is modulated by the triangular wave, then P′(*x*) = 2/π∙sgn(cos(*x*)) = ± 2/π. From Equation (13), *ν* is fluctuating ± 2*V*_p_*f*/*V*_π_ on the basis of *ν*_L_(*t*). The demodulation curve is the difference between *I*(*ν* = *ν*_L_(*t*) + 2*V*_p_*f*/*V*_π_) and *I*(*ν* = *ν*_L_(*t*) − 2*V*_p_*f*/*V*_π_). The corresponding expression of the demodulation curve can be expressed as

(16) $$\begin{array}{c} D\left(t\right)=I{|}_{\nu =\nu_{L}\left(t\right)+2V_{p}f/V_{\pi }}-I{|}_{\nu =\nu_{L}\left(t\right)-2V_{p}f/V_{\pi }}\\ =\left(1-k\right)\rho_{c}\sqrt{\rho_{c}\rho_{L}}\\ \;\cdot \{\frac{2\sqrt{k}\cos[2\pi (\nu_{L}\left(t\right)+\frac{2}{V_{\pi }}V_{p}f)\tau -\varphi_{0}]+\sqrt{\rho_{c}\rho_{L}}\left(1-k-2k\cos\varphi_{0}\right)}{1+k\rho_{c}\rho_{L}-2\sqrt{k\rho_{c}\rho_{L}}\cos[2\pi (\nu_{L}\left(t\right)-\frac{2}{V_{\pi }}V_{p}f)\tau ]}\\ \;-\frac{2\sqrt{k}\cos[2\pi (\nu_{L}\left(t\right)-\frac{2}{V_{\pi }}V_{p}f)\tau -\varphi_{0}]+\sqrt{\rho_{c}\rho_{L}}\left(1-k-2k\cos\varphi_{0}\right)}{1+k\rho_{c}\rho_{L}-2\sqrt{k\rho_{c}\rho_{L}}\cos[2\pi (\nu_{L}\left(t\right)+\frac{2}{V_{\pi }}V_{p}f)\tau ]}\}. \end{array}$$

**Figure 4:**
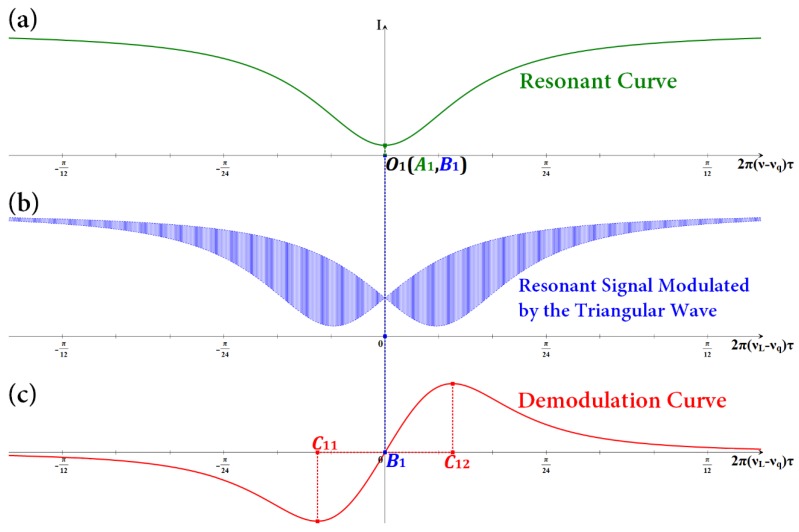
When *φ*_0_ = π, (**a**) simulated resonant curve, (**b**) simulated resonant signal modulated by the triangular wave, and (**c**) simulated demodulation curve.

**Figure 5:**
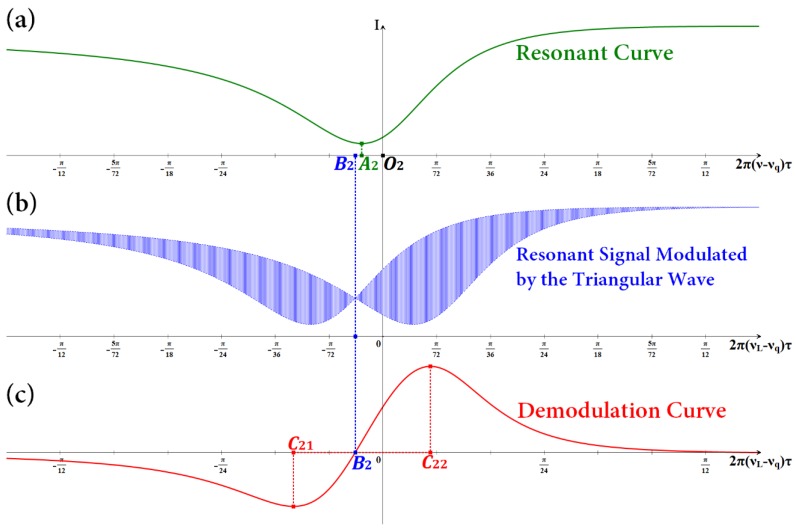
When *φ*_0_ = 0.9π, (**a**) simulated resonant curve, (**b**) simulated resonant signal modulated by the triangular wave, and (**c**) simulated demodulation curve can be obtained.

On the basis of the analysis above on the modulation and demodulation process, the resonant signal modulated by the triangular wave and the corresponding demodulation curve can be simulated. As shown in Figure 4, if the light passes through the cross port of coupler C2, then the phase is changed by π/2, that is, *φ*_0_ = π. Thus, the resonant curve is a signal with symmetry. According to Equation (7), when *ν* = *ν_q_*, cos 2π*ντ* takes the maximum value of 1, while |*E*_4_/*E*_1_|^2^ can get the minimum value. At the same time, the resonant signal modulated by the triangular wave and the corresponding demodulation curve are symmetrical. The abscissa *O*_1_ of the resonant frequency, the abscissa *A*_1_ of the lowest point of the resonant curve, and the abscissa *B*_1_ of the zero point of the demodulation curve completely coincide with each other. The linear region of the demodulation curve |*C*_11_*C*_12_| is also symmetrical, that is, |*C*_11_*B*_1_|=|*B*_1_*C*_12_|. The center frequency of the laser is finally locked at the zero point of the demodulation curve. Thus, the center frequency of the laser can be accurately locked at the resonant frequency of the light wave. As shown in Figure 5, if the light wave passes through the cross port of coupler C2, then the phase change is less than the ideal π/2, that is, *φ*_0_ ≠ π. Supposing that *φ*_0_ = 0.9π, then the resonant curve is a signal with asymmetry. At the same time, the resonant signal modulated by the triangular wave and the corresponding demodulation curve are asymmetrical. According to Equation (5), when *ν* = *ν_q_*, |*E*_4_/*E*_1_|^2^ cannot get the minimum value. The abscissa *O*_2_ of the resonant frequency, the abscissa *A*_2_ of the lowest point of the resonant curve, and the abscissa *B*_2_ of the zero point of the demodulation curve cannot coincide with each other. The linear region of the demodulation curve |*C*_21_*C*_22_| is also asymmetrical, that is, |*C*_21_*B*_2_|≠|*B*_2_*C*_22_|. Therefore, the central frequency of the laser cannot be accurately locked into the resonant frequency of the light wave, which affects the system open-loop bias and its stability and directly affects the angular velocity measurement accuracy.

## 4. Experimental Results and Discussion

**Figure 6:**
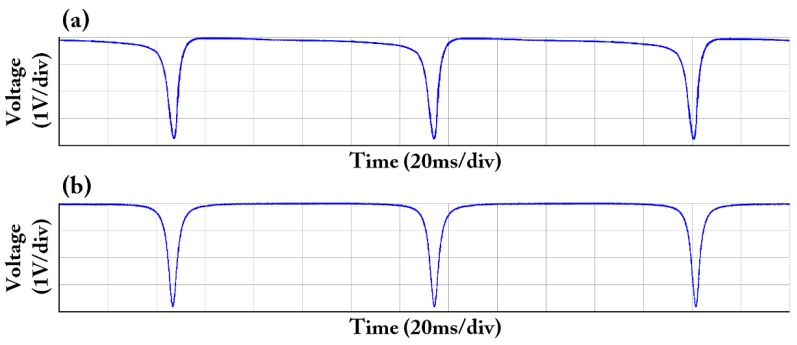
Measured resonant curve of the two different couplers connected to FRR. (**a**) C2_#1_; (**b**) C2_#2_.

As shown in Figure 1, we set up a RFOG system. To conduct a comparative experiment, we only change the coupler C2 connected to the FRR, while keeping the rest of the components of the RFOG system unchanged. We chose the couplers C2_#1_ and C2_#2_ from two different companies. The basic parameters of both couplers in the test sheet are similar. However, the normal mode loss difference of the coupler in the test sheets is not mentioned. A triangular wave with a frequency of 1 Hz and a peak-to-peak value of 1 V is produced by an arbitrary waveform generator to slowly change the frequency of the narrow linewidth semiconductor laser. The resonant curve observed from the oscilloscope when the coupler connected to the FRR is C2_#1_ is shown in Figure 6a. The asymmetry phenomenon in the experiment almost completely agrees with the asymmetry phenomenon shown in Figure 3b–d of the simulation results. We hypothesize that the asymmetry of this resonant curve is mainly caused by the normal mode loss difference of the coupler. Thus, we replace the coupler C2_#1_ connected with the FRR with the coupler C2_#2_, which solves the resonant curve asymmetry. The resonant curve that is observed from the oscilloscope is shown in Figure 6b. Thus, the resonant curve is a symmetrical signal. The experimental result is almost completely consistent with simulation result shown in Figure 3a.

**Figure 7:**
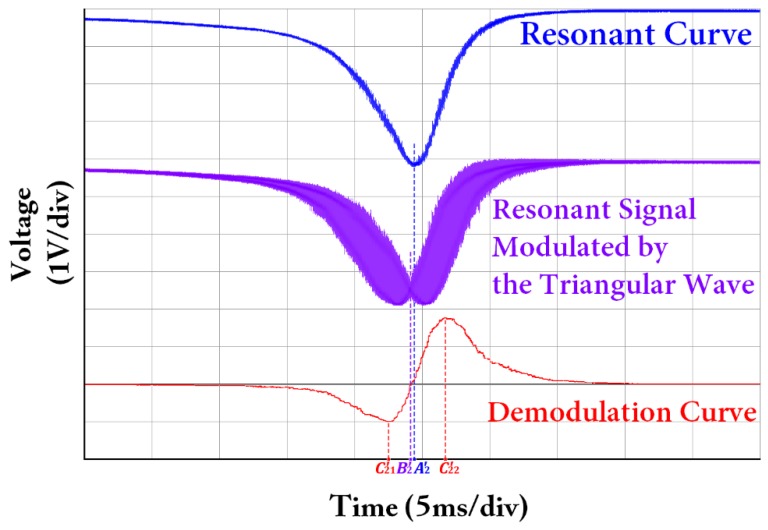
Measured resonant curve, resonant signal modulated by the triangular wave, and demodulation curve, when using coupler C2_#1_ connected to FRR.

**Figure 8:**
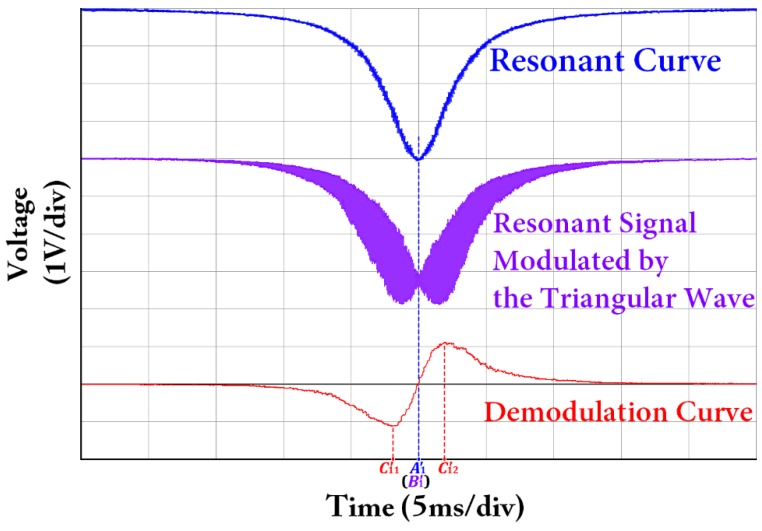
Measured resonant curve, resonant signal modulated by the triangular wave, and demodulation curve, when using coupler C2_#2_ connected to FRR.

The resonant curve, resonant signal modulated by the triangular wave, and the corresponding demodulation curve observed from the oscilloscope when the coupler connected to FRR is C2_#1_ are shown in Figure 7. Thus, the resonant curve is a signal with asymmetry. At the same time, the resonant signal modulated by the triangular wave and the corresponding demodulation curve are asymmetrical. The abscissa *A*_2_′ of the lowest point of the resonant curve and the abscissa *B*_2_′ of the zero point of the demodulation curve cannot coincide with each other. The linear region of the demodulation curve |*C*_21_′*C*_22_′| is also asymmetrical, that is, |*C*_21_′*B*_2_′| ≠ |*B*_2_′*C*_22_′|. Therefore, the central frequency of the laser cannot be accurately locked into the resonant frequency of the light wave, which affects the system open-loop bias and its stability, and directly affects the accuracy of the angular velocity measurement. The asymmetry phenomenon in the experiment almost completely agrees with the asymmetry phenomenon shown in Figure 5 of the simulation results. The resonant curve, the resonant signal modulated by the triangular wave and the corresponding demodulation curve observed from the oscilloscope after the coupler C2_#1_ connected with the FRR is changed with the coupler C2_#2_ are shown in Figure 8. The abscissa *A*_1_′ of the lowest point of the resonant curve and the abscissa *B*_1_′ of the zero point of the demodulation curve completely coincide with each other. The linear region of the demodulation curve |*C*_11_′*C*_12_′| is also symmetrical, that is, |*C*_11_′*B*_1_′| = |*B*_1_′*C*_12_′|. The center frequency of the laser is finally locked at the zero point of the demodulation curve. Thus, the center frequency of the laser can be accurately locked at the resonant frequency of the light wave. The experimental result is almost completely consistent with simulation result shown in Figure 4.

## 5. Conclusions

Resonance asymmetry phenomenon in RFOG is found in the experiment. In this paper, a new mathematic model is established. The influence of the normal mode loss difference of the coupler (the phase difference between the coupler cross port output optical field and direct port is less than the ideal π/2) on the symmetry of the resonant curve, the resonant signal modulated by the triangular wave, and the demodulation curve are all analyzed in detail. At the same time, it is proved that the asymmetry of the resonant curve will lead to the asymmetry of the resonant signal modulated by the triangular wave and the demodulation curve from the theoretical simulation and the experiment. The resonant curve asymmetry will affect the system open-loop bias and its stability, which directly affects the angular velocity measurement accuracy. Therefore, the analysis of the factor that affects the symmetry of the resonant curve is of great significance to improve the sensitivity of the gyroscope.
